# Nociceptor neurons shape antiviral immunity

**DOI:** 10.1186/s12974-026-03817-z

**Published:** 2026-04-21

**Authors:** Isaac Thomas, Anais Roger, Moutih Rafei, Katrina Gee, Sebastien Talbot, Sam Basta

**Affiliations:** 1 Department of Biomedical and Molecular Sciences, Queen’s University, Kingston, Canada; 2 Department of Pharmacology and Physiology, Universite de Montreal, Montreal, Canada; 3 Department of Physiology and Pharmacology, Karolinska Institutet, Solna, Sweden

**Keywords:** Neuro-immunology, Nociceptor neurons, Lymphocytic Choriomeningitis Virus (LCMV), Herpes Simplex Virus (HSV), Influenza A Virus (IAV), Infection, Adaptive immunity, Innate immunity, Inflammation, Antiviral immunity

## Abstract

The nervous and immune systems cooperate to protect the host, yet the contribution of sensory neurons to antiviral immunity remains incompletely defined. Nociceptor neurons do more than relay pain: they detect viral products and inflammatory cues through pattern-recognition receptors (PRRs), including Toll-like receptors (TLRs) and RIG-I-like receptors (RLRs), and respond to mediators such as type I interferons (IFNs), tumor necrosis factor (TNF), and interleukin-1β (IL-1β). Upon activation, these fibres release neuropeptides and neurotransmitters, including calcitonin gene-related peptide (CGRP) and substance P (SP), while sympathetic catecholamines provide a parallel neural input that shapes vascular tone, leukocyte trafficking, and effector programmes across tissues. Viral infection can also engage neuro-glial circuits in sensory ganglia and, in some settings, spread directly to the central nervous system (CNS), as described for lymphocytic choriomeningitis virus (LCMV), herpes simplex virus (HSV), and selected influenza A virus (IAV) strains. Here, we synthesize evidence that nociceptors shape antiviral immunity in a context-dependent manner rather than exerting uniform control. Nav1.8⁺ afferents restrain excessive inflammation while supporting dendritic cell (DC) priming of CD8⁺ T cells during cutaneous HSV-1 infection; vagal TRPV1⁺ neurons, a subset of Nav1.8⁺ nociceptors, promote disease tolerance during influenza by tuning lung myeloid responses; CGRP signaling drives T helper (Th) 1 differentiation during acute LCMV infection; and sympathetic adrenergic inputs deepen CD8⁺ T cell exhaustion during chronic infection. Defining these neuro-immune circuits may reveal therapeutic opportunities, but species differences, circuit heterogeneity, and the pleiotropic effects of shared mediators will need to be resolved before these insights can be translated to human disease.

## Introduction 

The peripheral nervous system (PNS) is divided into sensory and motor arms that convey afferent and efferent signals, respectively, between peripheral tissues and the CNS [1]. Within the somatosensory arm, specialised afferent neurons detect mechanical, thermal and chemical cues, supporting the perception of touch, pain, pressure, temperature and proprioception [1, 2]. Nociceptors, first responders to noxious stimuli, sense tissue damage, heat and inflammation [2]. They convey this information centrally via cell bodies housed in the dorsal root ganglia (DRGs), nodose ganglia (NG), and trigeminal ganglia (TG), with axonal projections that terminate in the spinal cord [2, 3]. These fibres extend peripherally to innervate nearly every organ, including the viscera, thereby forming a conduit for rapid bidirectional communication between injured tissues and the CNS [2, 3].

Nociceptors also infiltrate immune hubs with striking density [3–5]. In lymph nodes they enter through the hilus, track along blood vessels and arborise into the interstitium, accumulating in the medulla where newly activated lymphocytes emerge from the cortex [6]. This strategic positioning allows nociceptors to sample damage- and pathogen-associated molecular patterns (DAMPs and PAMPs), tightly modulate antigen trafficking through the lymphatic system, and interact directly with migrating immune cells [6, 7]. Extensive neuro-immune crosstalk ensues: immune cells secrete chemokines, lipid mediators and cytokines that bind cognate receptors on nociceptors [4], while neurons themselves express PRRs and TLRs that enable direct pathogen surveillance [4, 8]. In return, sensory fibres release neuropeptides and classical neurotransmitters that bind receptors on leukocytes, steering innate and adaptive responses throughout acute and chronic infection [3–5, 9, 10]. CGRP exemplifies the contextual breadth of neuronal influence across contexts. Although best known as a systemic vasodilator [11], CGRP can down-regulate HSV entry receptors on human Langerhans cells (LCs), limiting infection in vitro [12]. In acute LCMV infection, neuronal CGRP skews splenic and nodal CD4⁺ differentiation away from Th2 and toward antiviral Th1 profiles [13]. Collectively, these studies reveal how a single neuropeptide can dictate divergent immunological outcomes, underscoring the need to map neuro-immune circuits with cellular and pathogen resolution.

Throughout this review, we distinguish two non-exclusive scenarios. In one, neurotropic viruses directly infect neurons or neighboring glial cells. In the other, neurons are not productively infected but instead sense viral nucleic acids, DAMPs, or inflammatory mediators released from infected tissues. Separating these mechanisms is important because the same virus can engage both pathways, yet the relevant receptors, kinetics, and therapeutic implications differ substantially [4, 14].

## Viruses and the nervous system

### Neurotropism of LCMV

Arenaviruses are enveloped, negative-sense, single-stranded RNA (ssRNA) viruses that pass from their natural rodent hosts to humans chiefly through inhalation of aerosolised urine, faeces or saliva, or, less often, through breaches in the skin that contact contaminated material [15, 16]. Lassa virus, the most prevalent human arenavirus, causes Lassa fever, a haemorrhagic illness frequently complicated by encephalitis, ataxia and seizures—neurological sequelae that herald poorer survival [16, 17]. LCMV is the prototypical experimental arenavirus. In the house mouse (*Mus musculus*) it is transmitted vertically in utero or neonatally; infected animals remain persistently viraemic yet outwardly healthy [18–20]. Humans contract LCMV by inhaling contaminated aerosols or via broken skin [15]. Serosurveys estimate a prevalence of 2–15%, and roughly one-third of infections are silent [19, 21–27]. Person-to-person transmission is exceptional, but immunosuppressed transplant recipients who receive LCMV-positive grafts can develop a fulminant Lassa-like syndrome with hepatitis and multi-organ failure, underscoring the virus’s pathogenic potential in compromised hosts [21, 28–30].

LCMV displays striking tissue tropism, infecting immune cells, microglia, astrocytes, Schwann cells, fibroblasts, neurons and neural stem cells [31–40]. Cellular entry begins when the viral glycoprotein GP1 ligates α-dystroglycan (α-DG) or heparan sulphate on the plasma membrane [41, 42]. α-DG, which pairs with transmembrane β-dystroglycan, anchors cells to the extracellular matrix and guides immune cell trafficking and neuronal migration [43–45]. After receptor-mediated endocytosis, GP1 in late endosomes, having undergone N-linked glycosylation at residue N104 and exposure to acidic pH, switches to the lysosomal mucin CD164; the fusogenic GP2 subunit then drives membrane fusion, releasing the ribonucleoprotein complex into the cytosol [42]. The ambisense, bi-segmented genome comprises an S segment, encoding the nucleoprotein (NP) and the glycoprotein precursor (GPC), and an L segment, encoding the RNA-dependent RNA polymerase (L) and the matrix Z protein [46]. NP and L mRNAs are synthesised first and translated on free ribosomes [41, 46]. Each segment must then be replicated in the antigenomic (positive-sense) orientation before transcription of GPC and Z mRNAs ensues [46]. GPC enters the endoplasmic reticulum, where signal peptidase removes its N-terminal leader; site-1 protease in the Golgi subsequently cleaves the precursor into GP1 and GP2, which assemble as a heterodimer [41]. Newly synthesized NP, L, Z and glycoproteins complex with replicated genomic RNA, traverse the secretory pathway and bud from the plasma membrane, completing the replication cycle [41].

Experimental models in mice recapitulate this neuropathogenic capacity: LCMV readily infects Schwann cells, rich in α-DG, yet spares nociceptor neurons, whose α-DG expression is low [36]. Schwann cell infection is non-cytopathic; virions disrupt laminin-2 binding to α-DG within the basal lamina, compromising Schwann cell anchorage, derailing axonal myelination and producing neurological deficits including seizures, learning impairment and visual loss [36, 38]. Rambukkana and colleagues showed that the clone-13 strain fails to infect purified DRG neurons in vitro, whereas virtually all Schwann cells, either isolated or within DRG explants, become infected [36]. Although in vivo evidence is limited, circulating virus could conceivably infiltrate ganglia and target resident Schwann cells. Because LCMV is non-lytic, pathology primarily reflects the ensuing immune response; Schwann cell infection and meningeal inflammation together amplify neurological injury [20, 36].

### Neurotropism of HSV

Herpesviridae are a family of double-stranded DNA (dsDNA) viruses, including the highly neurotropic HSV type 1 (HSV-1) and HSV-2 [46]. These viruses pose a substantial threat to human health: seroprevalence studies indicate approximately 3.8 billion people globally under the age of 50 are infected with HSV-1, and 520 million people aged 15–49 are infected with HSV-2 [47]. While both types infect epithelial cells, HSV-1 primarily infects the oral mucosa, reflected in transmission via contact with cold sores or oral secretions from an infected individual [46]. Although HSV-1 has classically predominated in oral infection, it has increasingly been recognized as a cause of genital herpes, accounting for an approximate 2% annual increase in genital HSV-1 cases [46]. Conversely, HSV-2 is far more prevalent in the genital mucosa and is commonly transmitted through genital contact [46]. Infection of the oral mucosa can cause cold sores (blisters or ulcers), while HSV-2 and, to a lesser extent, HSV-1 manifest as genital herpes upon transmission to the genital mucosa, a disease characterized by fever, dysuria, itching or burning sensations, and lesions at the infection site [46].

HSV displays dual tropism: after replication in epithelial cells at the initial site, HSV-1 and HSV-2 can infect the sensory neurons innervating that site via viral glycoproteins gB, gC, gD and the gH/gL complex [47]. In sensory neurons, HSV gD binds the host receptor nectin-1, recruiting gB, gH and gL for secondary binding and ultimately driving membrane fusion at the peripheral axon terminals of TG or sacral DRG neurons [47–49]. Upon entry into the neuron, the capsid-associated tegument complex (CATC) is transported retrogradely along the axon to the neuronal cell body in the ganglion, mediated by dynein-dynactin recruitment to the CATC via the inner tegument protein pUL37 [50, 51]. Retrograde transport delivers HSV capsids to nuclear pores where, upon capsid rearrangement, the HSV genome enters the nucleus and establishes latency, allowing the viral genome to persist in the host cell nucleus, where only a small subset of viral genes are expressed [52]. 

### Neurotropism of IAV

As a major etiological agent of influenza (“the flu”), IAV belongs to the *Orthomyxoviridae* family, a group of negative-sense, segmented ssRNA viruses [53]. IAV spills over from its natural avian reservoirs to humans via direct contact with infected birds or their contaminated material (e.g., feces) [53]. Spreading among humans chiefly by airborne respiratory droplets, IAV infects around one billion people and causes 300,000–500,000 deaths each year through seasonal epidemics [54]. IAV binds and enters target cells through hemagglutinin (HA) binding to α2,6- or α2,3-linked sialic acids—a process heavily reliant on proteolytic cleavage of HA by the host proteases TMPRSS2 and HAT [55]. The restricted expression of TMPRSS2 and HAT to respiratory and gastrointestinal epithelium largely determines IAV tropism: infection is typically confined to these tissues, where viral replication in the lower respiratory tract can cause pneumonia and acute respiratory distress syndrome (ARDS) [56, 57]. In these pathologies, impaired lung function as well as loss of epithelium and alveolar structure result from lytic viral replication and the accompanying immune-mediated inflammation [57].

Interestingly, certain strains of IAV also show strong neurotropic potential [58]. Most notably, the highly pathogenic avian IAV strain H5N1 can directly infect peripheral neurons in vitro [59]. Jang and colleagues cultured mouse DRG neurons in microfluidic chambers separating cell bodies from distal axons; inoculation of the axonal compartment with H5N1 led to viral entry and retrograde transport, with viral antigen detectable in neuronal cell bodies 24 h post-infection (p.i.) [59]. Furthermore, Verzele et al. detected viral mRNA within vagal sensory ganglia at 4 and 6 days after H1N1 (swine-origin) IAV infection [60]. Notably, viral mRNA levels in vagal ganglia were reduced by administration of QX-314, a pharmacological silencer of TRPV1/TRPA1-expressing fibers [61]. Together, these data support the possibility that select IAV strains access sensory ganglia, although detection of ganglionic viral RNA does not by itself establish productive infection of nociceptors and could also reflect uptake of viral material or infection-associated trafficking within the ganglionic niche [60, 61].

In parallel with immune-mediated lung damage, IAV infection has been linked to neuropathies of the peripheral nerves, such as Guillain-Barré syndrome (GBS) and mononeuritis multiplex (MM), which provoke rapid-onset muscle weakness and paresthesia [62–64]. In GBS, IAV is thought to promote pathogenesis through molecular mimicry, wherein antibodies formed against IAV target gangliosides abundant on peripheral neuronal membranes and myelin sheaths, leading to the destruction of sensory and motor neurons [63]. Similarly in MM, autoantibodies and immune cells generated during IAV infection can mistakenly attack the endothelial cells lining the blood vessels that supply peripheral nerves [64]. A case report of a patient with MM-like symptoms found no typical causes aside from evidence of recent IAV infection [64]. Supporting a causal role for IAV, the patient did not respond to corticosteroids, but symptoms subsided after conservative management and oseltamivir treatment (an IAV antiviral) [64].

### Viruses in the CNS

Many viral infections initially replicate in the periphery and subsequently invade the CNS through diverse routes [14]. Viral infections of the brain and spinal cord often lead to virus-induced encephalitis and meningitis—pathologies largely mediated by the immune response—that can result in severe morbidity or even mortality [14]. Viral replication in neurons and glial cells triggers a robust type I IFN response: astrocytes secrete cytokines that recruit and activate microglia, which converge on infected foci and contribute to immune cell infiltration of the CNS [65]. Infected neurons, through MyD88-dependent signaling, release CXCL10 and CCL5, attracting CD8⁺ T cells and inflammatory monocytes whose perforin- and granzyme-mediated cytotoxicity is essential for viral clearance yet simultaneously drives neuropathology [65].

The laboratory-adapted LCMV Armstrong (Arm) strain, originally isolated from cerebrospinal fluid, is highly neurotropic [66]. Although it typically causes an acute infection, if unresolved, LCMV Arm can traverse from visceral organs to the CNS through three non-exclusive pathways: direct infection of brain microvascular endothelial cells (compromising the blood-brain barrier, BBB), carriage within leukocytes that cross an intact BBB, and, in congenital infections, direct entry into the CNS during development [38]. Once inside, viral antigen localizes mainly to the meninges, ependyma and choroid plexus, producing a classical lymphocytic meningitis [38, 67]. LCMV Arm shows a pronounced preference for glial over neuronal lineages. In mice, it colonises microglia and astrocytes and, to a lesser extent, neurons of the olfactory bulb and hippocampus [68]. Viral replication in these cells precipitates myelin loss, motor dysfunction and plaque-like inflammatory lesions, making glia-virus interactions central to LCMV neuropathogenesis [35]. Astrocyte destruction further weakens BBB integrity, raises intracranial pressure and exacerbates neurological symptoms [69].

In lifelong persistence resulting from vertical transmission of LCMV, virions perturb specialized neuronal circuits without overt cytolysis, selectively disabling differentiated cellular functions [70]. Molecularly, chronic infection suppresses both mRNA and protein for the growth-associated protein 43 (GAP-43), a marker of synaptic plasticity, thereby blocking the structural remodeling required for memory encoding [71]. In vivo, hippocampal GAP-43 protein levels fall in parallel with observed cognitive deficits: chronically infected mice perform poorly in spatial-temporal learning tasks and show diminished exploratory drive in a Y-maze [70, 72]. Kunz and colleagues extended these observations by showing that brains from persistently infected mice harbor widespread, non-destructive viral presence yet exhibit marked spatial-temporal learning deficits [73]. Transcriptomic profiling revealed 56 genes up-regulated, most encoding IFN-stimulated genes (ISGs) and their regulators [73]. Sustained ISG expression is known to impair neuronal excitability and synaptic function, suggesting that chronic IFN signaling—rather than direct cytolysis—drives the cognitive decline [73].

While the mechanisms are poorly understood, HSV can switch from latent to lytic infection in response to neuronal stress and associated histone modifications, producing new infectious virions that egress via anterograde transport toward peripheral sites to re-establish infection in the oral or genital mucosa [52]. Although less likely, the pseudounipolar structure of sensory neurons provides HSV a direct route to the CNS via central axonal branches that synapse in the spinal cord [14]. HSV-1 is particularly suited for CNS invasion; once latency is established in the TG, the virus can undergo anterograde transport along the central axon to reach trigeminal nuclei in the brainstem and thalamus, and subsequently the sensory cortex within the brain [48].

Acute HSV-1 infection in the CNS can cause herpes simplex encephalitis (HSE), a severe inflammatory disease of the brain driven by exuberant immune activation [48]. In HSE, glial cells such as microglia and astrocytes recognize conserved HSV-1 PAMPs via TLRs that are strategically positioned to detect both intra- and extracellular viral components [74]. Microglia express TLR3 in endosomes, enabling recognition of HSV-1 double-stranded RNA (dsRNA) intermediates [75, 76]. The resultant signaling cascade converges on IRF3, IRF7, and NF-κB, inducing type I IFNs and IL-1β and expediting antiviral responses against HSV-1 in the CNS [77]. Analogously, microglia and astrocytes detect extracellular HSV-1 glycoproteins (gH/gL and gB) through TLR2 on the cell surface [78]. TLR2 activation induces the production of cytokines and chemokines such as CCL2, which recruit monocytes and T cells to the CNS, further contributing to immune-mediated HSE pathogenesis [78]. Beyond endosomal TLRs, cytosolic DNA sensing through cGAS-STING is also likely to contribute to HSV-1 detection in glia and other CNS-resident cells, although its relative role in nociceptors remains less well defined than TLR3/TLR9 signaling [79].

Infection with the highly neurotropic H5N1 strain of IAV in humans often proves fatal and is associated with encephalitis, motor disturbances and even coma [59]. Using immunohistochemistry, Jang et al. mapped H5N1 dissemination in mice, detailing the spread from peripheral sites to the central neuraxis [59]. Beginning in the lung after intranasal inoculation, H5N1 moves systemically to the gastrointestinal tract, infecting mesenteric and Auerbach’s plexi of the enteric nervous system by day 2–3 p.i [59]. From here, H5N1 occupies DRGs before spreading to the CNS by day 3, initially replicating within brainstem nuclei [59]. By day 10, viral dissemination reaches all levels of the CNS—most notably the pons, thalamus, and cerebral cortex—infecting neurons in these regions before viral clearance occurs (viral NP became undetectable by day 21 in surviving mice) [59]. Within brain regions where H5N1 antigen was detected, microglia remained activated for at least 90 days p.i. (marked by Iba1 expression), indicating a prolonged neuroinflammatory response to IAV in the CNS [59].

## Sensory neuron detection of viral components and inflammation

**Fig. 1:**
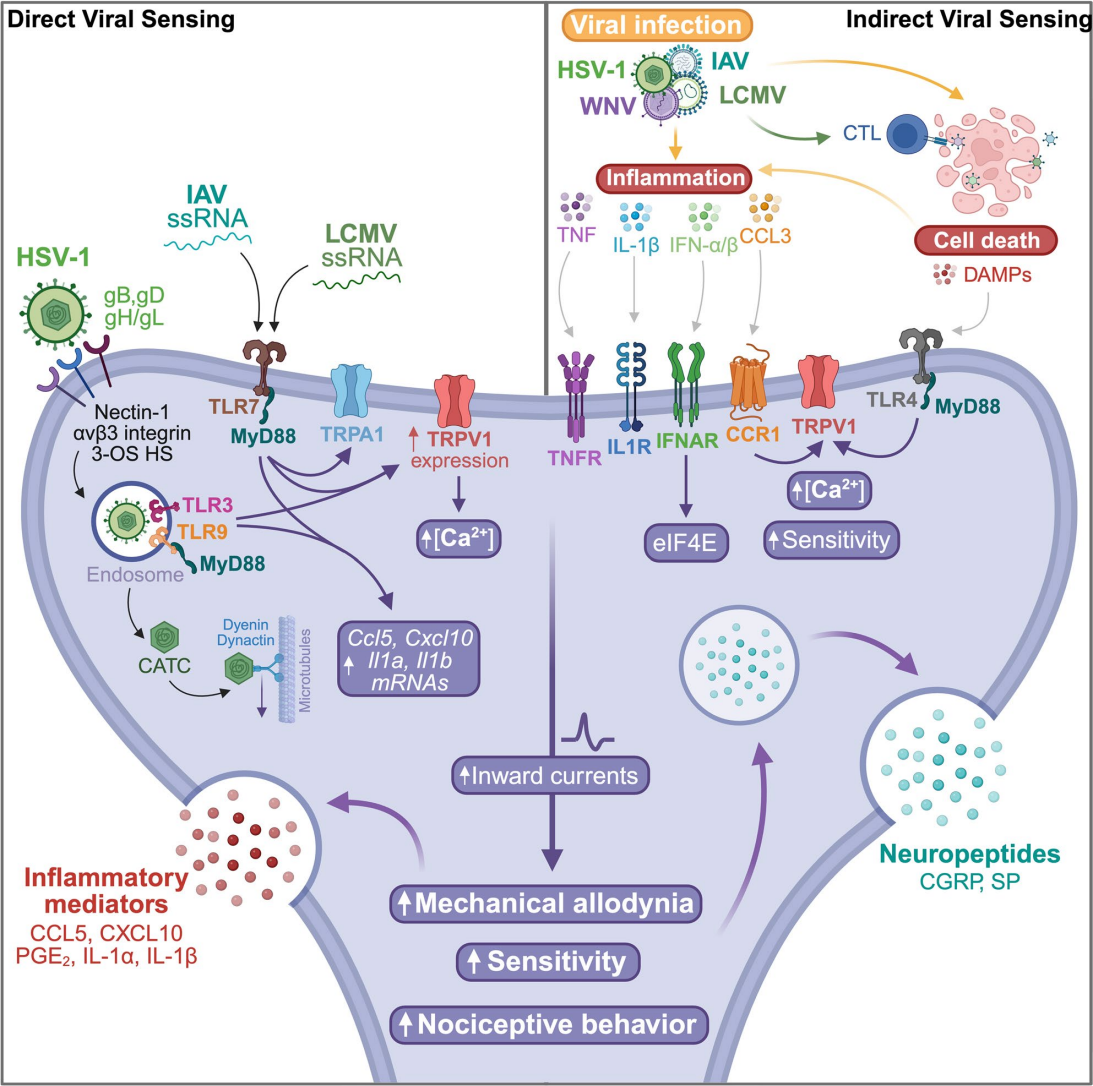
Direct and indirect viral sensing by nociceptors. Nociceptors express TLRs and cytokine/chemokine receptors that allow two broad modes of activation. On the left, direct sensing of viral components occurs through endosomal TLR3, TLR7, and TLR9, which detect dsRNA, ssRNA, and CpG DNA, respectively, and increase TRPV1/TRPA1-dependent excitability, calcium influx, and transcription of inflammatory mediators such as CCL5, CXCL10, IL-1α, IL-1β, and PGE2. HSV-1 can additionally enter sensory neurons via nectin-1 and related entry factors. On the right, indirect sensing follows infection-driven inflammation, during which cytokines, chemokines, and DAMPs released from infected or dying cells act on TNFR, IL1R, IFNAR, CCR1, and TLR4 to sensitize nociceptors. Examples include type I IFN signaling through IFNAR-JAK1/TYK2-MNK-eIF4E, CCL3 signaling through CCR1, and DAMP-mediated lowering of the TRPV1 activation threshold through TLR4. Together these inputs increase inward currents, mechanical allodynia, and nociceptive behavior while promoting neuropeptide release, including CGRP and SP, which can feed back on immune responses.

Even in the absence of productive neuronal infection, viruses provide a powerful lens through which to study neuro-immune crosstalk, because nociceptors can themselves act as sensors of both pathogens and inflammation. DRG, NG, and TG neurons express a broad repertoire of PRRs, including TLRs, enabling them to detect viral infection directly or indirectly even when viruses do not replicate within nociceptors [4, 8]. TLR4, which recognizes viral envelope proteins and infection-associated DAMPs [80, 81], has been reported to associate with TRPV1 through the TIR domain of TLR4; ligand engagement, including by DAMPs released during cell death, lowers the activation threshold of TRPV1 [82–84]. Although the mechanism underlying TLR4-TRPV1 coupling remains unclear, the resulting inward currents through TRPV1 increase neuronal excitability and trigger CGRP exocytosis, which can shape a wide range of context-dependent immune outcomes [82–84] (Fig. 1). TLR7, which detects ssRNA [85], is similarly functionally linked to the cation channel TRPA1 [86]. Although the mechanism is again unresolved, activation of TLR7 by specific microRNAs containing a GUUGUGU motif evokes inward currents and spontaneous action potentials, producing rapid nocifensive behavior in mice [84, 86, 87] (Fig. 1). TLR3 and TLR9 are likewise expressed in nociceptors and are strategically localized to endosomes, where they can detect dsRNA, a common viral replication byproduct, and unmethylated CpG DNA, respectively [8]. Activation of either receptor further enhances TRPV1 sensitivity to capsaicin, highlighting the capacity of viral sensing pathways to both prime inflammatory programs and increase nociceptor excitability [8] (Fig. 1). Consistent with this, the adaptor MyD88, required for nearly all intracellular TLR signaling, is abundant in nociceptors; conditional deletion of MyD88 blunted inflammatory hyperalgesia and limited macrophage ingress into sensory ganglia, demonstrating that neuronal TLR signaling can shape both pain and neuroinflammation [88].

Viruses such as LCMV and IAV may engage nociceptors either indirectly through TLR4, via infection-induced DAMPs such as oxidized phospholipids and HMGB1, or directly through TLR7 via viral ssRNA, thereby increasing neuronal excitability [89–91] (Fig. 1). Nociceptors also express cytosolic RLRs, which detect 5′-triphosphate ssRNA generated during viral replication [92, 93]. RIG-I ligation promotes aggregation of mitochondrial antiviral-signaling protein (MAVS) on mitochondria, leading to activation of TBK1/IKKε, phosphorylation of IRF3, and induction of type I IFNs (IFN-α/β), which rapidly act on innate immune cells to establish an antiviral state [94, 95]. LCMV subverts this pathway at two levels: its zinc-binding Z protein binds MAVS to block downstream signaling, while its NP sequesters IRF3, preventing nuclear translocation and dampening IFN transcription [41, 96]. Even so, nociceptors continue to express surface IFN-α/β receptors (IFNAR1/2); IFNAR engagement activates JAK1/TYK2 and, in addition to canonical STAT1/2 signaling, can engage a noncanonical MNK-eIF4E pathway that further sensitizes nociceptors and evokes mechanical allodynia [97] (Fig. 1). Whether nociceptor activation is driven primarily by direct viral sensing or by secondary inflammatory cues likely varies across tissues and over time.

HSV and its components, particularly following endocytic uptake, can activate TLR3 and TLR9 [79]. In sensory neurons, activation of these receptors induces both mRNA and protein expression of proinflammatory mediators, including CCL5, CXCL10, IL-1α, and IL-1β, demonstrating a direct neuronal contribution to local inflammatory recruitment programs [8] (Fig. 1). TLR3 or TLR9 activation also increases TRPV1 expression and calcium influx, suggesting that HSV can directly enhance nociceptor excitability [8] (Fig. 1). Beyond these endosomal pathways, cytosolic DNA-sensing pathways that converge on STING, potentially including the cGAS-STING axis, are plausible contributors to HSV-1 detection. STING signaling and the resulting production of type I IFNs in nociceptors have been shown to critically shape nociception [98]. Indeed, HSV-1 dsDNA elicits pain through STING-dependent signaling in nociceptors, likely through a mechanism distinct from canonical TBK1 recruitment and NF-κB/IRF3 activation [99]. This response requires TRPV1 expression in nociceptors, although the basis for TRPV1 involvement and the identity of the relevant upstream dsDNA sensor(s) remain largely unknown [99].

Neuroglial circuits add a second layer of modulation to these neuron-intrinsic sensing pathways. Schwann cells and satellite glia express PRRs and can respond directly to viral motifs [100]. For example, exposure of Schwann cells to HIV-1 gp120 induces CXCL1, which recruits macrophages to nociceptor fibers and amplifies hypersensitivity [101]. During HSV or West Nile virus (WNV) infection, microglia activated either by direct infection or by sensing viral components release proinflammatory cytokines such as TNF and IL-1β [102, 103] (Fig. 1).

LCMV infection likewise induces sustained expression of TNF and IL-1β. One study documented progressive increases in both cytokines in the brain and peripheral organs during acute LCMV infection [104]. Even athymic nude mice, which lack the CD8⁺ T cell response that drives classic choriomeningitis, upregulated TNF and IL-1β, indicating an innate source [104]. In parallel, LCMV-specific CD8⁺ T cells secreted the chemokine CCL3, which appeared to exacerbate pathology [105, 106]. Nociceptors expressing the tetrodotoxin-resistant sodium channel Nav1.8, including a subset marked by TRPV1 expression, respond robustly to TNF, IL-1β, and CCL3, underscoring their capacity for indirect viral sensing and suggesting a likely role in viral pathogenesis and the associated immune response [107, 108] (Fig. 1). IL-1β provides a clear example of direct neuronal modulation: it activates p38 MAPK, shifts persistent sodium currents toward depolarization, lowers firing thresholds, and heightens heat and mechanical hypersensitivity [109] (Fig. 1). Although the precise mechanism remains unclear, CCL3 signals through CCR1, which is often co-expressed with TRPV1, to enhance TRPV1 sensitivity to capsaicin and anandamide; accordingly, recombinant CCL3 shortened hot-plate latency in vivo [108] (Fig. 1). Activated nociceptors, in turn, release CGRP and SP (Fig. 1), dilating the microvasculature, increasing endothelial permeability, facilitating leukocyte extravasation, and enhancing cytokine production by DCs, macrophages, and mast cells, thereby establishing a feed-forward loop of neurogenic inflammation [4]. Together, these findings support a model in which antiviral immune responses sensitize and activate nociceptors, which then feed back onto local inflammation and immune polarization to shape disease outcomes.

## Nociceptor neurons regulate antiviral immunity

Neuro-immune crosstalk now occupies a central place in our understanding of pathogen control because neurons can fine-tune innate and adaptive immune cell behavior in ways that depend on anatomical site, viral persistence, and the identity of the sensory or autonomic fibers engaged. These bidirectional interactions unfold in peripheral lymphoid organs, barrier tissues, and—once activated lymphocytes traverse the BBB—within the CNS itself. Their impact is therefore highly context dependent, with polarity dictated by the virus encountered, the tempo of antigen clearance, the neuronal subset engaged, and the type of perturbation used to interrogate the circuit. Accordingly, we use terms such as “shape” or “modulate” for most phenotypes and reserve stronger causal language for receptor-defined mechanisms.

Sensory olfactory neurons in fish display an intriguing first line of defense against the fish rhabdovirus infectious hematopoietic necrosis virus (IHNV) [110]. In their model, Sepahi et al. report a direct interaction between the IHNV G protein and the TrkA receptor on a subset of olfactory sensory neurons in teleost fish, which induces proinflammatory responses and drives the infiltration of a unique CD8⁺ T cell population to the olfactory organ, where viral replication occurs [110]. The total ablation of this subset of neurons correlated with increased susceptibility to IHNV, underlining the potential importance of sensory neurons as an early line of antiviral defense [110].

**Fig. 2:**
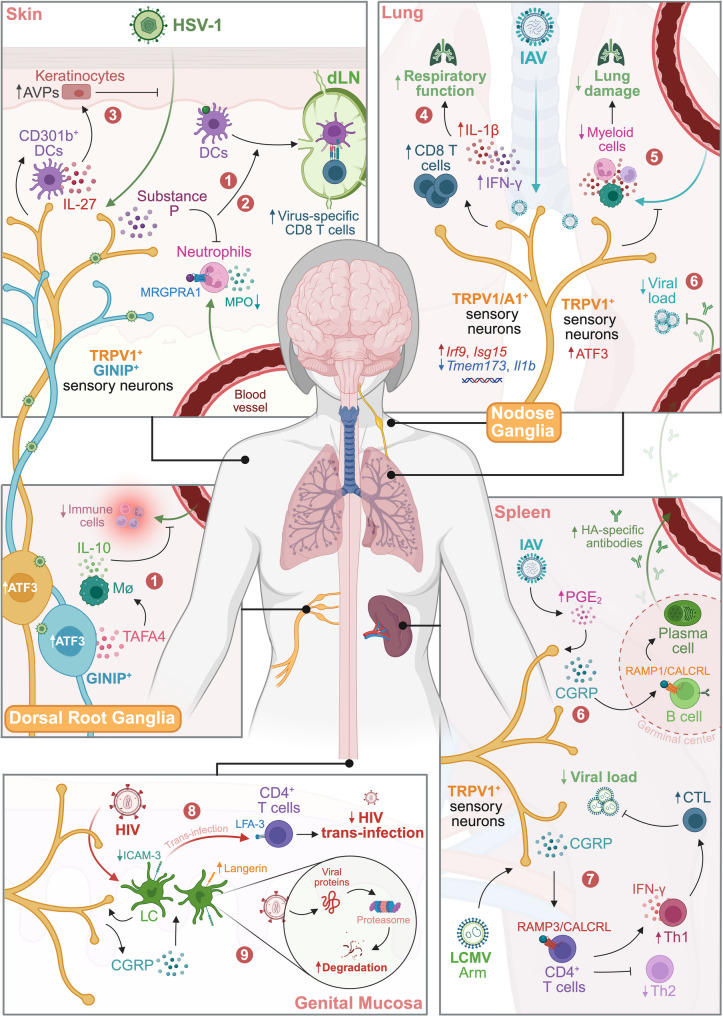
Sensory neurons modulate antiviral immunity and inflammation across tissues. Sections 1 and 2 depict cutaneous HSV-1 infection: the broader Nav1.8⁺ nociceptor afferent population constrained neutrophil influx, limited lesion severity, and preserved DC priming of virus-specific CD8⁺ T cells in dLNs [111], while a subsequent study resolved partially overlapping subsets and showed that TRPV1⁺ neurons released SP to restrain neutrophil recruitment through MRGPRA1 in the skin, whereas GINIP⁺ neurons released TAFA4 in DRGs to promote IL-10-dependent resolution of post-viral inflammation [112]. Section 3 shows that TRPV1⁺ cutaneous neurons promoted IL-27-dependent induction of antiviral proteins in wounded skin, reducing HSV-1 susceptibility [113]. Sections 4 and 5 summarize influenza studies in which pulmonary/vagal sensory circuits limited disease severity by supporting IFN-competent myeloid responses and CD8⁺ T cell accumulation, while vagal TRPV1⁺ neurons restrained excessive lung myeloid infiltration and damage [61, 114]. Section 6 shows that splenic TRPV1⁺ nociceptors increased CGRP release in response to PGE2 and signaled through CALCRL-RAMP1 on B cells to support germinal center responses, plasma cell output, and HA-specific antibody production during H1N1 infection [115]. Section 7 depicts acute LCMV infection, in which nociceptor-derived CGRP signaled through RAMP3-CALCRL on CD4⁺ T cells to promote Th1 differentiation and strengthen antiviral IFN-γ production [13]. Sections 8 and 9 depict human mucosal tissue, where CGRP released from sensory neurons acted on LCs to increase langerin, reduce ICAM-3, and divert HIV-1 toward proteasomal degradation, thereby limiting trans-infection of CD4⁺ T cells [118, 120].

The lymph node provides a compelling example. In cutaneous HSV-1 infection, Nav1.8⁺ nociceptor afferents helped constrain inflammatory responses and supported DC priming of CD8⁺ T cells in skin-draining lymph nodes (dLNs) of mice [111] (Fig. 2). Genetically ablating these fibers unleashed excessive neutrophil recruitment in the skin and amplified inflammatory cytokine and chemokine production (notably IL-1β, IL-6, TNF, IFN-β, GM-CSF, CXCL1, and CCL3) [111]. This immune dysregulation in nociceptor-deficient mice corresponded with large necrotic lesions at the site of cutaneous HSV infection, independent of viral replication or clearance [111]. Critically, neuronal restraint of neutrophil infiltration was linked to conventional DC and T cell responses, as the absence of Nav1.8⁺ signaling blunted the priming and expansion of CD8⁺ T cells during HSV-1 infection—an effect reversed by neutrophil depletion [111]. Because Nav1.8-directed strategies target a broad nociceptor-enriched population rather than a single molecularly defined circuit, these data support a protective neuronal role but do not by themselves identify the dominant effector mediator [111].

A subsequent study further resolved this broad nociceptor contribution by separating partially overlapping TRPV1⁺ and GINIP⁺ subsets from the broader Nav1.8⁺ population interrogated previously [112] (Fig. 2). Roger and colleagues found that two distinct sensory neuron subsets became activated by HSV-1 and independently limited tissue inflammation in the DRG and skin, respectively, without measurably altering viral load [112]. GINIP⁺ sensory neurons released TAFA4 in response to HSV-1, acting through an IL-10 axis to prevent excessive immune cell infiltration into innervating DRGs after viral clearance, as ablation of TAFA4 or administration of IL-10-neutralizing antibodies led to significant increases in monocytes, macrophages, and neutrophils at day 8 p.i [112]. Conversely, TAFA4 was not produced in the skin after HSV-1 infection and therefore had no detectable effect on cutaneous immunity or zosteriform lesions [112]. By contrast, TRPV1⁺ sensory neurons limited lesion severity in the skin and supported adaptive immune responses in the dLN, as specific ablation by the vanilloid toxin resiniferatoxin (RTX) caused a massive influx of neutrophils into the skin by day 5 p.i. and severe inflammatory lesions [112]. RTX-treated mice also exhibited reduced numbers of DC subsets in the dLN at day 8 p.i., corresponding to impaired virus-specific CD8⁺ T cell expansion [112]. This balance between inflammation and adaptive immunity depended on SP release in the skin, as *Tac1* knockout in wild-type mice recapitulated the effects of TRPV1⁺ ablation, whereas exogenous SP rescued RTX-mediated defects, decreasing HSV-1 skin lesions and neutrophil influx while restoring the CD8⁺ T cell response [112]. SP acted directly on neutrophils via its receptor MRGPRA1 to dampen neutrophil migration into the skin [112]. MRGPRA1-deficient neutrophils accumulated excessively in HSV-1-infected skin, while neutrophil depletion or inhibition of neutrophil-derived myeloperoxidase restored DC numbers and the CD8⁺ T cell response in RTX-treated mice [112]. Together, these data identified tissue-specific TAFA4-IL-10 and SP-MRGPRA1 axes that promoted disease tolerance during HSV-1 infection [112].

Nociceptors also played a direct role against invading HSV-1 at the skin barrier (Fig. 2). Lei et al. found that acute skin wounding in mice and humans induced multiple genes encoding immune signaling molecules and antiviral proteins, most prominently *OAS2* and *OASL2* [113]. This response depended on TRPV1⁺ cutaneous nociceptors, as TRPV1 ablation reversed the effect whereas capsaicin enhanced it [113]. Because AVP-producing CD301b⁺ DCs responded to IL-27, the authors examined the role of TRPV1⁺ nociceptors in this pathway and found that genetic ablation similarly decreased *Il27p28* induction in CD301b⁺ cells, correlating with a marked decrease in OAS2 expression at the wound edge [113]. They extended this model to HSV-1 infection using skin explants from *Trpv1*-/- mice and found that loss of TRPV1 signaling rendered the skin significantly more susceptible to HSV-1 infection [113]. Taken together, these findings delineated a neuro-immune axis in which TRPV1⁺ nociceptors promoted IL-27-dependent antiviral protein induction in wounded skin, implicating their role in strengthening cutaneous resistance to HSV-1 [113].

In the lung, vagal TRPV1⁺ sensory neurons also appeared to promote disease tolerance during IAV infection by restraining myeloid-cell-driven immunopathology [114] (Fig. 2). Almanzar et al. reported that pharmacological or genetic ablation of TRPV1⁺ and Nav1.8⁺ sensory neurons markedly reduced survival in IAV-infected mice without measurably altering viral load [114]. Loss of TRPV1⁺ fibers increased lung damage and was accompanied by elevated bronchoalveolar concentrations of IL-6, G-CSF, IL-4, IL-13, LIF, and IL-11, together with an altered IFN response that was consistent with unrestrained inflammation [114]. RTX-treated mice also exhibited increased infiltration of neutrophils, monocytes, and monocyte-derived macrophages by day 7 p.i., along with impaired IFN signaling in monocytes and altered neutrophil states [114]. TRPV1⁺ vagal neurons up-regulated the injury-induced transcription factor ATF3 during infection, which was consistent with neuronal activation but did not, on its own, distinguish direct viral sensing from secondary inflammatory activation [114]. Myeloid cells were functionally important because antibody-mediated depletion of these cells, or restoration of IFN signaling, improved survival [114]. These data therefore supported a protective neuronal circuit while also underscoring the interpretive limits of broad ablation strategies [114].

The spleen painted a comparable picture. Thoracic TRPV1⁺ sensory neurons coursed with splenic blood vessels and densely innervated germinal centers to promote humoral immunity in mice [115] (Fig. 2). Wu and colleagues used NP-KLH immunization to show that prostaglandin E2 (PGE2) accumulation after immunization activated TRPV1⁺ sensory neurons, increased calcium influx, and triggered CGRP release [115]. Authentic H1N1 IAV infection also increased splenic PGE2 and CGRP, suggesting that IAV may engage a similar pathway of antigen-induced nociceptor activation [115]. CGRP activated cAMP signaling in B cells through a CALCRL-RAMP1 axis. Consistent with this, ablation of TRPV1⁺ nociceptors or deletion of CALCRL in B cells impaired germinal center B cell formation, splenic plasma cell generation, and virus-specific antibody production [115]. Conversely, activation of these nociceptors by dietary capsaicin enhanced HA-specific IgG responses to IAV infection, reduced viral load and immune cell infiltration in the lungs, limited weight loss, and improved survival [115]. In this setting, the neuronal effector axis was therefore defined more precisely than in broad ablation models, linking splenic PGE2-TRPV1 activation to CGRP-CALCRL-RAMP1 signaling in B cells [115].

Remarkably, different subsets of peripheral sensory neurons can alter host immunity in opposite ways during infection with the same IAV strain. In mice infected with IAV H1N1, PGE2 levels were also markedly elevated in the airways and acted on EP3 receptors on a subset of GABRA1⁺ glossopharyngeal sensory neurons innervating the upper respiratory tract [116]. PGE2 engagement of EP3 activated these neurons, elicited sickness behaviors such as reduced food intake and mobility, and reduced overall survival; genetic ablation of this neuronal subset or targeted knockout of EP3 reversed this phenotype [116]. Together with the lung-protective vagal circuits described below, these findings illustrate that the net contribution of sensory neurons during influenza depends strongly on anatomical compartment, behavioral output, and the phase of disease being measured.

A complementary study focused on pulmonary vagal sensory neurons innervating the lungs rather than on the broader TRPV1⁺ or Nav1.8⁺ populations interrogated above [61] (Fig. 2). Pharmacological silencing of pulmonary vagal fibers with QX-314 increased expression of some host-defense genes (for example *Tmem173* and *Il1b*) but decreased expression of IFN-response genes (*Irf9* and *Isg15*) in vagal ganglia [61]. This corresponded with lower IL-1β and IFN-γ levels, as well as reduced numbers of macrophages and CD8⁺ T cells in the lungs of infected mice [61]. Consequently, mice with silenced vagal sensory neurons lost more weight and showed worse respiratory function, consistent with more severe disease [61]. Considered together with RTX- and genetic-ablation studies, these data suggest that apparently discordant influenza phenotypes likely reflect differences in neuronal subset identity, anatomical targeting, timing, and experimental modality rather than a simple binary protective-versus-pathogenic role for nociceptors [61, 114, 116].

Murine LCMV infection further illustrated how neuropeptides and classical neurotransmitters can reprogram T cell responses. In acute infection with the Arm strain (given intraperitoneally), CGRP released from neurons binds its heterodimeric receptor (RAMP3-CALCRL) on naïve T cells [13]. Engagement with RAMP3-CALCRL elevates intracellular cAMP, activates CREB and ATF3, augments STAT1 expression and drives robust IFN-γ production, thereby skewing differentiation toward the Th1 lineage [13] (Fig. 2). Virus-specific CD4⁺ Th1 cells and CD8⁺ cytotoxic T lymphocytes expanded vigorously; mice lacking RAMP3 or CALCRL generate smaller effector pools, produce less cytokine and harbor higher viral loads [13].

By contrast, persistent infection with the clone 13 strain produced the opposite outcome. Chronic antigen exposure engaged a sympathetic stress axis in which terminally exhausted CD8⁺ T cells upregulated the β₁-adrenergic receptor (ADRB1) and localized near sympathetic axons that released the catecholamines norepinephrine and epinephrine [117]. Signaling through ADRB1 activated adenylate cyclase, increased intracellular cAMP, and ultimately suppressed T cell proliferation and cytokine production, thereby reinforcing the exhausted state [117]. Consistent with this, genetic ablation of catecholamine production, pharmacological catecholamine depletion, or direct ADRB1 blockade reduced PD-1 and TIM-3 expression, restored cytokine production, and improved T cell proliferation [117].

Beyond neuronal modulation over immune responses and inflammation as a whole, CGRP-secreting peripheral neurons have also been reported to potentiate antiviral mechanisms at the cellular level. Upon mucosal entry, HIV-1 became rapidly internalized by LCs, which directly contacted peripheral sensory neurons releasing CGRP [118, 119]. This was followed by trans-infection of local CD4⁺ T cells, initiating classical HIV pathology [118]. Ganor and colleagues found that in LCs from human donors, CGRP signaling increased langerin expression, decreased expression of specific integrins including ICAM-3, and activated NF-κB [118]. Collectively, these perturbations inhibited LC-T cell conjugate formation, promoted CCL3 secretion, and limited HIV-1 trans-infection of CD4⁺ T cells [118] (Fig. 2). A follow-up study further showed that CGRP shifted HIV-1 degradation pathways in mucosal LCs from an endolysosomal to a proteasomal fate, resulting in more rapid degradation and reduced virion availability for transfer to CD4⁺ T cells [120] (Fig. 2). HIV-1-infected individuals exhibited loss of cutaneous innervation and reduced plasma CGRP levels, a phenotype rescued by antiretroviral therapy, suggesting that establishment of HIV-1 trans-infection and dissemination may be favored when nociceptor tone is reduced [118]. These observations provide an important human example of a neuro-immune pathway with direct antiviral consequences at a post-entry step in the viral lifecycle.

Besides CGRP and catecholamines, other neuropeptides broaden the regulatory spectrum. SP released from TRPV1⁺ fibers can enhance vascular permeability, act as a leukocyte chemoattractant, and amplify IFN-γ and TNF production by T cells and macrophages, thereby supporting Th1 immunity [121, 122]. Paradoxically, SP also mobilizes CD301b⁺ DCs to dLNs, where they bias differentiation toward Th2; the net outcome therefore hinges on the relative strength of these opposing pathways [123].

Vasoactive intestinal peptide (VIP) and pituitary adenylate cyclase-activating polypeptide (PACAP) drive immune responses toward Th2: they reprogram macrophages to secrete IL-4 and IL-5 via up-regulation of B7.2, suppress IFN-γ-producing cells and dampen microglial pro-inflammatory output [124]. Somatostatin (SST), produced by specialized inhibitory interneurons, further tempers immune responses by limiting immunoglobulin synthesis, restraining T cell proliferation and inhibiting IFN-γ release [125]. Intriguingly, LCMV Arm replicates within SST-positive neurons and reduces SST transcripts without overt neuronal damage [126], raising the possibility that viral suppression of this inhibitory pathway could favor viral clearance or, alternatively, contribute to immunopathology. This breadth of neuropeptide-mediated modulation of the immune response exemplifies the intricate interplay between the nervous and immune systems that can influence viral infection outcomes in the host.

## Conceptual challenges, controversies, and translational relevance

Several unresolved issues complicate the synthesis of findings across viral systems. Productive neuronal infection should not be conflated with neuronal activation. HSV-1 and selected IAV strains can infect sensory neurons under defined conditions, whereas many antiviral phenotypes more likely reflect neuronal sensing of viral nucleic acids, cytokines, chemokines, or DAMPs in the absence of productive neuronal infection [8, 48, 59, 61]. This distinction is mechanistically important because the receptors engaged, the kinetics of activation, and the most tractable therapeutic nodes are likely to differ between these scenarios. It is also critical for interpretation: detection of viral RNA in ganglia, or induction of neuronal stress programmes such as ATF3, is consistent with direct nociceptor infection but does not, on its own, establish it. For example, ganglion-resident glial cells may themselves become infected or sense nearby infection, thereby initiating neuronal activation, stress responses, or downstream immune effects. Glial cells should therefore be considered active intermediaries in these circuits rather than passive support cells.

Apparently conflicting influenza studies likely reflect genuine biological heterogeneity rather than simple inconsistency. Upper-airway GABRA1⁺ glossopharyngeal neurons promoted sickness behaviors that reduced survival, whereas pulmonary vagal TRPV1⁺ circuits and splenic TRPV1⁺ fibers promoted disease tolerance or humoral immunity [61, 114–116]. These outcomes differed by anatomical compartment, neuronal subset, phase of infection, and readout (behavior, myeloid restraint, IFN competence, or antibody production). Experimental design also matters: RTX ablation, Nav1.8-Cre strategies, genetic deletion, and transient QX-314 silencing interrogate overlapping but non-identical neuronal populations and can differentially perturb baseline tissue homeostasis, vascular regulation, breathing, feeding, and stress physiology. Broad perturbation models therefore support the conclusion that neurons matter, but they often stop short of identifying a single causal effector axis.

Translational extrapolation remains limited because most mechanistic evidence derives from mice, yet human and murine nociceptor repertoires, tissue innervation density, and immune organization are not identical. Human-relevant data nevertheless suggest that the core concepts are likely conserved: HSV uses nectin-1 to enter human sensory neurons, CGRP limits HSV and HIV-1 interactions with human LCs, and viral infections are clinically linked to sensory dysfunction, neuropathy, and neuroinflammatory sequelae [12, 49, 62, 118]. Therapeutic manipulation of these pathways will therefore require caution. For example, blocking CGRP signaling might be attractive for pain control or to limit HIV-1 trans-infection, yet the same intervention could plausibly weaken protective CGRP-dependent Th1 polarization in acute LCMV or antibody responses during influenza, likely favouring opportunistic secondary infections [13, 115, 118].

Several emerging areas are poised to sharpen this field. Single-cell and spatial transcriptomic approaches should help resolve which neuronal, glial, stromal, and immune subsets participate in viral circuits at barrier tissues, ganglia, and lymph nodes [6, 11, 65]. The notion of discrete neuro-immune synapse-like contacts, the active role of satellite glia, Schwann cells, microglia, and astrocytes as intermediaries, and the modifying effects of metabolic state, sex, and prior inflammatory history all remain underexplored but are likely to explain part of the context dependence emphasized throughout this review [65, 100].

## Conclusion

Together, the evidence reviewed here supports a model in which viral outcomes are shaped not only by canonical immune pathways but also by tightly localized neuro-immune circuits in barrier tissues, ganglia, lymphoid organs, and the CNS. Across HSV-1, IAV, LCMV, and HIV-1, nociceptor-derived mediators influenced leukocyte recruitment, DC priming, T cell polarization, humoral immunity, and disease tolerance. The common principle is not that neurons uniformly protect or uniformly worsen disease, but that defined circuits tune antiviral responses according to tissue context, infection stage, and ligand-receptor pairing, informing targeted therapeutic strategies to prevent adverse implications.

The next phase of the field will require circuit-resolved and human-relevant validation. Spatial mapping, single-cell profiling, and receptor-specific perturbation strategies should be used to separate direct viral sensing from indirect inflammatory activation, identify the immune cells that receive neuronal signals, and test how glia, metabolic state, and sex modify these interactions over time. Such precision will be essential if neuro-immune pathways are to be therapeutically targeted without sacrificing antiviral protection or essential sensory function. 

## Data Availability

No datasets were generated or analysed during the current study.

## Abbreviations

ADRB1: β1-adrenergic receptor
α-DG: Alpha-dystroglycan
ARDS: Acute respiratory distress syndrome
Arm: Armstrong (LCMV Armstrong strain)
ATF3: Activating transcription factor 3
AVP: Antiviral protein(s)
B7.2: B7.2 costimulatory molecule (CD86)
BBB: Blood-brain barrier
CALCRL: Calcitonin receptor-like receptor
cAMP: Cyclic adenosine monophosphate
CATC: Capsid-associated tegument complex
CCL3: C-C motif chemokine ligand 3 (MIP-1α)
CCL5: C-C motif chemokine ligand 5 (RANTES)
CCR1: C-C chemokine receptor 1
CD164: Cluster of differentiation 164
CD301b: Cluster of differentiation 301b
CD4: Cluster of differentiation 4
CD8: Cluster of differentiation 8
CGRP: Calcitonin gene-related peptide
CNS: Central nervous system
CpG: Cytosine-phosphate-guanine (CpG) DNA motif
CREB: cAMP response element-binding protein
CTL: Cytotoxic T cell / cytotoxic T lymphocyte
CXCL1: C-X-C motif chemokine ligand 1 (CXCL1/KC)
CXCL10: C-X-C motif chemokine ligand 10 (IP-10)
DAMP: Damage-associated molecular pattern(s)
DC: Dendritic cell(s)
dLN: Draining lymph node(s)
DNA: Deoxyribonucleic acid
DRG: Dorsal root ganglion
dsDNA: Double-stranded DNA
dsRNA: Double-stranded RNA
eIF4E: Eukaryotic translation initiation factor 4E
EP3: Prostaglandin E receptor 3
G-CSF: Granulocyte-colony-stimulating factor
GABRA1: GABA-A receptor α1 subunit (gamma-aminobutyric acid type A receptor alpha-1 subunit)
GAP-43: Growth-associated protein 43
gB: (HSV) glycoprotein B
GBS: Guillain-Barré syndrome
GC: Germinal centre / germinal center
gC: (HSV) glycoprotein C
gD: (HSV) glycoprotein D
gH: (HSV) glycoprotein H
GINIP: Gαi-interacting protein (G protein signaling regulator)
gL: (HSV) glycoprotein L
GM-CSF: Granulocyte macrophage colony-stimulating factor
GP1: (LCMV) glycoprotein 1
gp120: (HIV-1) envelope glycoprotein 120
GP2: (LCMV) glycoprotein 2
GPC: Glycoprotein precursor
HA: Hemagglutinin
H1N1: Influenza A virus subtype H1N1
H5N1: Influenza A virus subtype H5N1
HAT: Human airway trypsin-like protease (host protease that cleaves influenza HA)
HIV: Human immunodeficiency virus
HIV-1: Human immunodeficiency virus type 1
HMGB1: High mobility group box 1
HSE: Herpes simplex encephalitis
HSV: Herpes simplex virus
HSV-1: Herpes simplex virus type 1
HSV-2: Herpes simplex virus type 2
IAV: Influenza A virus
Iba1: Ionized calcium-binding adapter molecule 1 (AIF1; microglial marker)
ICAM-3: Intercellular adhesion molecule 3
IFN: Interferon
IFN-α/β: Type I interferons (interferon-α and interferon-β)
IFN-β: Interferon-beta
IFN-γ: Interferon-gamma
IFNAR: Interferon-α/β receptor (type I interferon receptor)
IFNAR1/2: Interferon-α/β receptor subunits 1 and 2
IHNV: Infectious hematopoietic necrosis virus
IKKε: IκB kinase epsilon (IKKi)
IgG: Immunoglobulin G
IL-1α: Interleukin-1 alpha
IL-1β: Interleukin-1 beta
IL-10: Interleukin-10
IL-11: Interleukin-11
IL-13: Interleukin-13
IL-27: Interleukin-27
IL27p28: Interleukin-27 subunit p28
IL-4: Interleukin-4
IL-5: Interleukin-5
IL-6: Interleukin-6
IRF3: Interferon regulatory factor 3
IRF7: Interferon regulatory factor 7
IRF9: Interferon regulatory factor 9
ISG: Interferon-stimulated gene
ISG15: Interferon-stimulated gene 15
JAK1: Janus kinase 1
KLH: Keyhole limpet hemocyanin
L: (LCMV) RNA-dependent RNA polymerase (L protein)
LC: Langerhans cell
LCMV: Lymphocytic choriomeningitis virus
LIF: Leukemia inhibitory factor
MAPK: Mitogen-activated protein kinase
MAVS: Mitochondrial antiviral-signaling protein
CCL2: C-C motif chemokine ligand 2 (legacy name MCP-1)
MM: Mononeuritis multiplex
MNK: MAPK-interacting kinase (Mnk1/2)
MPO: Myeloperoxidase
MRGPRA1: Mas-related G-protein–coupled receptor A1
mRNA: Messenger RNA
MyD88: Myeloid differentiation primary response 88
Nav1.8: Voltage-gated sodium channel Nav1.8 (tetrodotoxin-resistant)
NF-κB: Nuclear factor kappa-B
NG: Nodose ganglion
NP: Nucleoprotein
NP-KLH: Nitrophenyl–keyhole limpet hemocyanin (NP conjugated to KLH immunogen)
OAS2: 2′-5′-oligoadenylate synthetase 2
OASL2: 2′-5′-oligoadenylate synthetase-like 2
p38: p38 mitogen-activated protein kinase (p38 MAPK)
PACAP: Pituitary adenylate cyclase-activating polypeptide
PAMP: Pathogen-associated molecular pattern(s)
PD-1: Programmed cell death protein 1
PGE2: Prostaglandin E2
p.i.: Post-infection
PNS: Peripheral nervous system
PRR: Pattern-recognition receptor
QX-314: QX-314 (quaternary lidocaine derivative; membrane-impermeant sodium-channel blocker)
RAMP1: Receptor activity-modifying protein 1
RAMP3: Receptor activity-modifying protein 3
RIG-I: Retinoic acid–inducible gene I (cytosolic RNA sensor; DDX58)
RLR: RIG-I-like receptor(s)
RNA: Ribonucleic acid
RTX: Resiniferatoxin
S: (LCMV) small genome segment (S segment)
SP: Substance P
SST: Somatostatin
ssRNA: Single-stranded RNA
STAT1: Signal transducer and activator of transcription 1
STAT1/2: Signal transducer and activator of transcription 1 and 2
Tac1: Tachykinin 1 (gene encoding substance P)
TAFA4: TAFA4 / FAM19A4 (chemokine-like protein)
TBK1: TANK-binding kinase 1
TG: Trigeminal ganglion
Th1: T helper 1
Th2: T helper 2
TIM-3: T cell immunoglobulin and mucin-domain containing-3
TLR: Toll-like receptor
TLR2: Toll-like receptor 2
TLR3: Toll-like receptor 3
TLR4: Toll-like receptor 4
TLR7: Toll-like receptor 7
TLR9: Toll-like receptor 9
TMEM173: Transmembrane protein 173 (STING)
TMPRSS2: Transmembrane serine protease 2
TNF: Tumor necrosis factor
TrkA: Tropomyosin receptor kinase A (high-affinity nerve growth factor receptor)
TRPA1: Transient receptor potential ankyrin 1
TRPV1: Transient receptor potential vanilloid 1
TYK2: Tyrosine kinase 2
VIP: Vasoactive intestinal peptide
WNV: West Nile virus
Z: (LCMV) matrix Z protein
pUL37: (HSV) tegument protein UL37 (inner tegument protein)
